# Loss-of-function variants in *ITSN1* confer high risk of Parkinson’s disease

**DOI:** 10.1038/s41531-024-00752-9

**Published:** 2024-08-15

**Authors:** Astros Th. Skuladottir, Vinicius Tragante, Gardar Sveinbjornsson, Hannes Helgason, Arni Sturluson, Anna Bjornsdottir, Palmi Jonsson, Vala Palmadottir, Olafur A. Sveinsson, Brynjar O. Jensson, Sigurjon A. Gudjonsson, Erna V. Ivarsdottir, Rosa S. Gisladottir, Arni F. Gunnarsson, G. Bragi Walters, Gudrun A. Jonsdottir, Thorgeir E. Thorgeirsson, Gyda Bjornsdottir, Hilma Holm, Daniel F. Gudbjartsson, Patrick Sulem, Hreinn Stefansson, Kari Stefansson

**Affiliations:** 1 deCODE genetics/Amgen Inc., Reykjavik, Iceland; 2 Faculty of Medicine, University of Iceland, Reykjavik, Iceland; 3 Heilsuklasinn Clinic, Reykjavik, Iceland; 4 Department of Geriatric Medicine, Landspitali University Hospital, Reykjavik, Iceland; 5 Department of Internal Medicine, Landspitali University Hospital, Reykjavik, Iceland; 6 Faculty of Icelandic and Comparative Cultural Studies, University of Iceland, Reykjavik, Iceland; 7 Faculty of Engineering and Natural Sciences, University of Iceland, Reykjavik, Iceland

**Keywords:** Genetic association study, Rare variants, Parkinson's disease, Parkinson's disease

## Abstract

Parkinson’s disease (PD) is a debilitating neurodegenerative disorder and its rising global incidence highlights the need for the identification of modifiable risk factors. In a gene-based burden test of rare variants (8647 PD cases and 777,693 controls) we discovered a novel association between loss-of-function variants in *ITSN1* and PD. This association was further supported with burden data from the Neurodegenerative Disease Knowledge Portal and the Accelerating Medicines Partnership Parkinson’s Disease Knowledge Platform. Our findings show that Rho GTPases and disruptions in synaptic vesicle transport may be involved in the pathogenesis of PD, pointing to the possibility of novel therapeutic approaches.

## Introduction

Parkinson’s disease (PD) is a complex neurodegenerative disorder with a large effect on individuals and society^1^. The disorder is mainly characterized by death of dopaminergic neurons in the pars compacta of the substantia nigra^1,2^ as a result of aberrant α-synuclein accumulation in Lewy bodies^3,4^, dysfunction of mitochondria, lysosomes or vesicle transport, and synaptic transport issues^1^.

PD develops from a complicated interplay between genetics and environment^1^. Genome-wide association studies (GWAS) have associated over 90 common variants with PD^5^. In GWAS, associations with rare variants often go undetected due to the low number of carriers. However, high-impact variants in coding regions often undergo negative selection and thus, tend to be rare. Unlike common variants, rare variants are often located within coding regions and may have substantial effects. The use of large-scale whole-genome sequence (WGS) data enable a more comprehensive search for rare variants. Combining variants predicted to cause a loss of gene function increases the statistical power^6,7^ and may allow us to pinpoint genes involved in the pathogenesis of PD. Previously, a meta-analysis found that the burden of rare variants in nine genes associated with PD using 7184 PD cases, 6701 proxy cases, and 51,650 controls of European ancestry^8^.

**Fig. 1:**
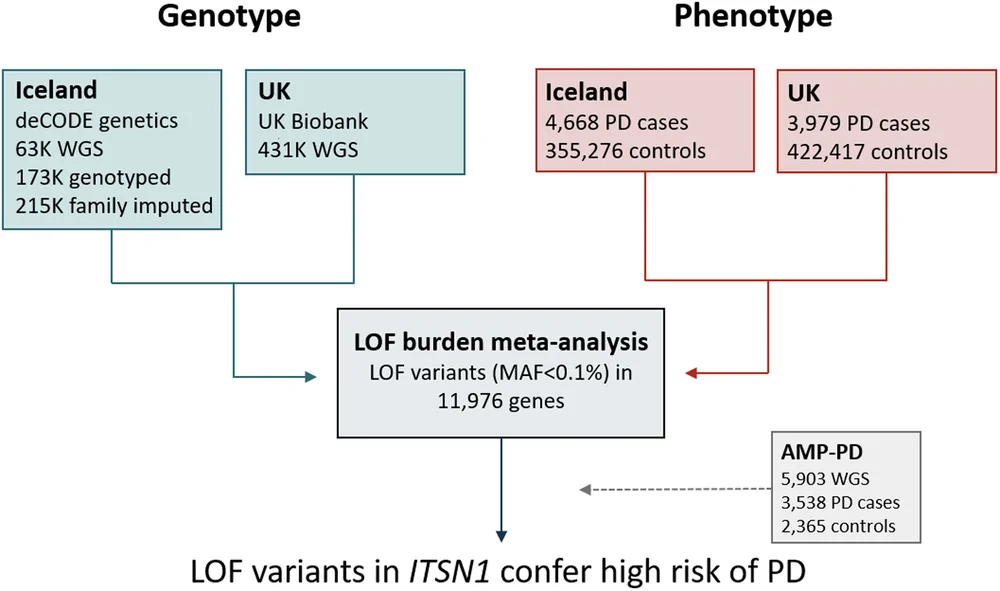
Overview of study design and summary of results.

In a gene-based burden test, we collapsed rare variants (MAF < 0.1%) predicted to cause loss-of-function (LOF) as estimated with transcript effect (LOFTEE)^9^ using WGS data. We tested for association between PD and burden of LOF in 11,976 genes (*P* < 0.05/11,976 = 4.2 × 10^−6^) in Iceland and the UK (Supplementary Data 1 and Supplementary Fig. 1). We combined the results in a meta-analysis using 8647 PD cases and 777,693 controls (Fig. 1, Supplementary Data 2 for demographics) of European descent, of which 62% have been WGS and the rest chip-genotyped and imputed. For the top finding, we included the Accelerating Medicines Partnership Parkinson’s Disease dataset (AMP-PD) in the meta-analysis, adding 3538 PD cases and 2365 controls, in total 12,185 PD cases and 780,058 controls.

Rare LOF variants in *ITSN1* (Cumulative Variant Frequency [CVF] = 0.012% in Iceland, CVF = 0.013% in the UK, and CVF = 0.10% in AMP-PD) were associated with a large effect on the risk of PD (OR = 7.3, 95% Confidence Interval [CI] = 3.5–15.2, *P* = 1.5 × 10^−7^, Table 1). The effects in the datasets were similar (*P*-het = 0.86). In Iceland, we found five LOF variants in *ITSN1* and the association was mainly driven by the two most common ones, a splice acceptor (chr21:33781560-A, c.1597-1 G > A, MAF = 0.006%, *P* = 0.028, OR = 6.1) and a stop-gain variant (rs1569244177-A, p.Trp944Ter, MAF = 0.005%, *P* = 0.034, OR = 10.0, Supplementary Data 3). In the UK, we found 64 LOF variants in *ITSN1* (Supplementary Data 3). Among the 154 carriers, 13 (8.4%) were diagnosed with PD compared to 1.1% of individuals diagnosed with PD in the two population-based datasets. In the AMP-PD dataset (*N* = 5903 WGS), we found six carriers of six rare LOF variants (MAF < 0.1%, Supplementary Data 3), thereof 5 with PD.

**Table 1:** Association results for burden of LOF in *ITSN1*

Gene	Dataset	Variants (*N*)	CVF cases/controls (%)	*N* carriers (cases)	*P* value	OR (95% CI)	*P*-het
*ITSN1*	Meta-analysis	75	0.15/0.018	160 (18)	1.5 × 10^−7^	7.3 (3.5–15.2)	0.86
	Iceland	5	0.11/0.010	41 (5)	2.4 × 10^−3^	7.5 (2.0–27.4)	
	UK	64	0.20/0.025	113 (8)	2.2 × 10^−5^	7.8 (3.0–20.1)	
	AMP-PD	6	0.14/0.042	6 (5)	0.41	3.35 (0.37–158.2)	

We find support for the association (*P* = 8.9 × 10^-6^) in a comparable LOFTEE rare variant (MAF < 0.1%) burden test including over 750,000 individuals from the UK dataset (*N* ~ 450 K), All of Us (*N* ~ 250 K), and Massachusetts General Brigham Biobank (*N* ~ 53 K) - results shown in the Neurodegenerative Diseases Knowledge Portal^10^.

The youngest *ITSN1* carrier with PD, diagnosed at 27 years of age, did not have variants in other genes described as pathogenic by OMIM^11^. Neither did any of the other PD diagnosed carriers. The average age at onset was 8 years earlier among *ITSN1* carriers (*P* = 0.022) than non-carriers (62 years, SD = 12.2 vs 70 years, SD = 9.8, respectively, Supplementary Data 2 for each dataset).

We note that *ITSN1* is intolerant of LOF variants (probability of LOF intolerance [pLI] = 1), according to the gnomAD database^12^, and both heterozygous LOF and missense variants in the gene have been associated with neurodevelopmental disorders^13^.

*ITSN1* encodes Intersectin-1, a cytoplasmic membrane-associated protein involved in two main pathways; (1) actin cytoskeleton rearrangements through the Rho GTPase cycle^14,15^ and (2) clathrin-mediated endo- and exocytosis, including the synaptic vesicle cycle^14,16^.

One of the pathological hallmarks of PD is the early death of dopaminergic neurons in the pars compacta of substantia nigra^1^. The growth and degeneration of dopaminergic neurons depend on the actin cytoskeleton which is regulated by small GTPases of the Rho family^15,17^, encompassing Rho, Rac, and CDC42 subfamilies. ITSN1 acts as a guanine nucleotide exchange factor that specifically activates CDC42^18^, Cell Division Control Protein 42, and can, thus, modulate regulation of the actin cytoskeleton. Several findings suggest the involvement of CDC42 in PD. Downregulation of *CDC42* has been observed in various brain regions, including substantia nigra, in postmortem brain tissues from PD patients^19^. In PD mouse models, Cdc42 signaling is decreased in the caudate and putamen, accompanied by impairments in motor coordination and cognitive function^20^ and *Cdc42* knockout mice display similar phenotype as parkinsonian mice^20^.

Aggregation of α-synuclein in Lewy bodies is another pathological hallmark of PD^1^. Numerous proteins have been shown to interact with α-synuclein, including CDC42^21^. Activating Rho GTPases is followed by substantial reduction in α-synuclein expression in dopaminergic neurons^17^ and neurite extension^22^.

In conclusion, we report a novel gene associated with PD and suggest that the loss of *ITSN1* function may be involved in the pathogenesis of PD in at least one of three ways; (1) Inactive CDC42 and its downstream pathway results in degeneration of dopaminergic neurons, (2) inactive CDC42 cannot regulate the vesicle exocytosis of α-synuclein, and (3) disrupted synaptic vesicle transport, a known feature in the pathogenesis of PD^1^, via clathrin-mediated endo- and exocytosis. Thus, direct modulation of CDC42 or its upstream regulator, ITSN1, could be exploited as therapeutic avenue for PD.

The results for all genes tested are available (Supplementary Data 1) and will hopefully become a useful resource in future PD studies.

## Methods

### Study sample and ethics statement

Icelandic PD cases were identified by ICD-10 code G20 and ICD-9 code 332.0 from medical records, filed from 1985 to 2023, through collaboration with physicians at Landspitali—National University Hospital in Reykjavik, the Registry of Primary Health Care Contacts, and the Registry of Contacts with Medical Specialists in Private Practice. The data in this study was approved by the National Bioethics Committee (NBC, VSN-17-142; VSNb2017060004/03.01) following review by the Icelandic Data Protection Authority. All genotyped participants signed a broad informed consent allowing the use of their samples and data in projects at deCODE genetics approved by the NBC. Personal identifiers of the participants’ data were encrypted in accordance with the regulations of the Icelandic Data Protection Authority.

The UKB resource consists of extensive phenotype and genotype data from ~500,000 participants, who enrolled in the study between 2006 and 2010 throughout the UK and were aged 40 to 69 years at recruitment^23^. PD cases were identified by ICD-10 code G20 in General Practice clinical event records (Field ID 42040), ICD-10 code G20 and ICD-9 code 332.0 in UK hospital inpatient data (Field ID 41270 and 41271), and self-report in non-cancer self-reported illness records (Field ID 20002). This study was conducted under application number 42256. All participants provided an informed consent for the use of their genotype data and the link to electronic health records. The North West Research Ethics Committee reviewed and approved the UKB protocol (ref. 06/MRE08/65).

### Genotyping

The genomes of 58,346 Icelanders were WGS^24,25^ using GAIIx, HiSeq, HiSeqX, and NovaSeq Illumina technology to a mean depth of at least 38×. Joint variant calling was performed with Graphtyper (v2.7.1)^26,27^. Roughly 155,000 Icelanders were genotyped (of which all were WGS) using various Illumina SNP arrays^24,25^. The genotypes were long-range phased^28^, allowing for improved genotype calls using haplotype sharing information. Subsequently, familial imputation of genotypes in first and second degree relatives was used to increase the sample size^29^.

In the UKB, 431,079 white British/Irish individuals (identified by PCA analyses)^23^ were WGS using Illumina NovaSeq sequencing machines at deCODE for 214,548 individuals and Wellcome Trust Sanger Institute for 216,531 individuals. The average genome-wide sequencing coverage was 32.4x and joint variant calling was performed using GraphTyper (v.2.7.5)^26,27^. The protocol for a preliminary release of the WGS of the UK Biobank dataset has been described in detail^30^.

### Variant quality control and annotation

Only high-quality sequence variants were considered for selection. To estimate the quality of the sequence variants we regressed the alternative allele counts on the depth conditioned on the genotypes reported by GraphTyper^26,27^. For a well-behaving sequence variant, the mean alternative allele count should be 0 for a homozygous genotype, depth/2 for a heterozygous genotype, and depth for homozygous alternative genotype. Assuming no sequencing or genotyping errors, the expected value of alternative allele count should be depth conditioned on the genotype, represented by an identity line (slope 1, intercept 0). Deviations from this line suggest a spurious or somatic sequence variant. We filtered variants with slope less than 0.5. Additionally, Graphtyper assigns each variant a score (AAscore), predicting the probability that it is a true positive. We only included variants with AAscore > 0.8.

Variant Effect Predictor (VEP)^31^ was used to attribute predicted consequences to the variants sequenced in the two datasets. LOF variants were classified as those predicted as start-lost, stop-gain, stop-lost, splice donor, splice acceptor, or frameshift. LOF variants with MAF < 0.1% were further evaluated by LOFTEE^9^, which determined which high-confidence LOF variants were used in this study.

### Association and meta-analysis

We applied logistic regression under an additive model to test for association between gene-based burden of LOF variants and PD where the disease status was the dependent variable and genotype counts were the independent variable. To compute two-sided *P* values, we used likelihood ratio test. Individuals were coded 1 if they carry any LOF variants in the autosomal gene being tested and 0 otherwise. In the Icelandic dataset, we adjusted for sex, current age or age at death, county of origin (equivalent to principal components), blood sample availability, and an indicator function for the overlap of the lifetime of the individual with the time span of phenotype collection. In the UK dataset, we adjusted for sex, age, the first 20 principal components and three variables indicating sequencing batches, to remove batch effects. We used LD score regression intercepts^32^, 1.14 for Iceland and 1.00 for the UK, to adjust the χ^2^ statistics and avoid inflation due to cryptic relatedness and stratification, using a set of 1.1 million variants. *P*-values were calculated from the adjusted χ^2^ results.

Meta-analysis was performed on the summary results from the two datasets when available, using a fixed-effects inverse variance weighted method^33^, in which the datasets were allowed to have different population frequencies for alleles and genotypes but were assumed to have a common odds ratio (OR) and weighted with the inverse of the variance of the effect estimate derived from the logistic regression. The significance threshold was based on the number of genes tested (Bonferroni significance). In a random-effects method, a likelihood ratio test was performed to test the heterogeneity of the effect estimate in the datasets; the null hypothesis is that the effects are the same in the two datasets and the alternative hypothesis is that the effects differ between datasets.

### Additional datasets

The Neurodegenerative Disease Knowledge Portal (NDKP) framework is an open platform developed by a team of scientists and software engineers at the Broad Institute of MIT and Harvard. The NDKP includes results generated via consortia based science focusing on neurodegenerative diseases such as ALS, PD, and Alzheimer’s disease. More information on https://ndkp.hugeamp.org/.

The Accelerating Medicines Partnership^®^ (AMP^®^) program is a public-private partnership between the National Institutes of Health (NIH), multiple biopharmaceutical and life sciences companies, and non-profit organizations. Release 3.0 cohorts include the Michael J. Fox Foundation for Parkinson’s Research (MJFF) Parkinson’s Progression Marker Initiative (PPMI), The National Institute of Neurological Disorders and Stroke (NINDS) BioFIND study, Harvard Biomarkers Study (HBS), the NINDS Parkinson’s Disease Biomarkers Program (PDBP), the MJFF LRRK2 Cohort Consortium (LCC), the NINDS Study of Isradipine as a Disease Modifying Agent in Subjects With Early Parkinson Disease, Phase 3 (STEADY-PD3), the MJFF and NINDS Study of Urate Elevation in Parkinson’s Disease, Phase 3 (SURE-PD3), and the Global Parkinson’s Genetics Program (GP2). WGS was performed by Macrogen and the Uniformed Services University of Health Sciences using the Illumina HiSeq XTen sequencer with samples coming from whole blood. The data were processed using GATK Best Practices guidelines set by the Broad Institute’s joint discovery pipeline and elaborated on elsewhere^34^. Variant annotations were generated on the joint genotyped variants with VEP. All individuals were of European ancestry as confirmed by principal component analysis using HapMap3 European ancestry populations. The *P* value in Table 1 was generated with a two-sided Fisher’s Exact Test in R.

## Supplementary information

Supplementary Figure

Supplementary Data

## Data Availability

Summary data of the gene-burden meta-analysis are in Supplementary Data 1.

## References

[1] Kalia, L. V. & Lang, A. E. Parkinson’s disease. *Lancet* **386**, 896–912 (2015). 10.1016/S0140-6736(14)61393-3 25904081

[2] Dickson, D. W. et al. Neuropathological assessment of Parkinson’s disease: refining the diagnostic criteria. *Lancet Neurol.* **8**, 1150–1157 (2009). 10.1016/S1474-4422(09)70238-8 19909913

[3] Spillantini, M. G. et al. α-Synuclein in Lewy bodies. *Nature* **388**, 839–840 (1997). 10.1038/42166 9278044

[4] Polymeropoulos, M. H. et al. Mutation in the α-synuclein gene identified in families with Parkinson’s disease. *Science* **276**, 2045–2047 (1997). 10.1126/science.276.5321.2045 9197268

[5] Nalls, M. A. et al. Identification of novel risk loci, causal insights, and heritable risk for Parkinson’s disease: a meta-analysis of genome-wide association studies. *Lancet Neurol.* **18**, 1091–1102 (2019). 10.1016/S1474-4422(19)30320-5 PMC8422160 31701892

[6] Li, B. & Leal, S. M. Methods for detecting associations with rare variants for common diseases: application to analysis of sequence data. *Am. J. Hum. Genet.* **83**, 311–321 (2008). 10.1016/j.ajhg.2008.06.024 PMC2842185 18691683

[7] Lee, S., Abecasis, G. R., Boehnke, M. & Lin, X. Rare-variant association analysis: study design and statistical tests.*Am. J. Hum. Genet.* **95**, 5 (2014). 10.1016/j.ajhg.2014.06.009 PMC4085641 24995866

[8] Makarious, M. B. et al. Large-scale rare variant burden testing in Parkinson’s disease. *Brain* **146**, 4622–4632 (2023). 10.1093/brain/awad214 PMC10629770 37348876

[9] Karczewski, K. J. et al. The mutational constraint spectrum quantified from variation in 141,456 humans. *Nature* **581**, 434–443 (2020). 10.1038/s41586-020-2308-7 PMC7334197 32461654

[10] Neurodegenerative Diseases Portal. Available at: https://ndkp.hugeamp.org/ accessed 4 January 2024.

[11] Online Mendelian Inheritance in Man, OMIM®. Johns Hopkins University, Baltimore, MD. MIM Number: PS168600. World Wide Web URL: https://omim.org/ Available at: https://www.omim.org/ accessed 3 November 2023.

[12] ITSN1 | gnomAD v4.0.0 | gnomAD. Available at: https://gnomad.broadinstitute.org/gene/ENSG00000205726?dataset=gnomad_r4 accessed 20 November 2023.

[13] Bruel, A. L. et al. ITSN1: a novel candidate gene involved in autosomal dominant neurodevelopmental disorder spectrum. *Eur. J. Hum. Genet.* **30**, 111–116 (2021). 10.1038/s41431-021-00985-9 PMC8738743 34707297

[14] Pechstein, A., Shupliakov, O. & Haucke, V. Intersectin 1: a versatile actor in the synaptic vesicle cycle. *Biochem. Soc. Trans.* **38**, 181–186 (2010). 10.1042/BST0380181 20074056

[15] Hall, A. & Lalli, G. Rho and Ras GTPases in axon growth, guidance, and branching. *Cold Spring Harb. Perspect. Biol.* **2**, a001818 (2010). 10.1101/cshperspect.a001818 PMC2828272 20182621

[16] Katsu, M. et al. MicroRNA expression profiles of neuron-derived extracellular vesicles in plasma from patients with amyotrophic lateral sclerosis. *Neurosci. Lett.* **708**, 134176 (2019). 10.1016/j.neulet.2019.03.048 31173847

[17] Zhou, Z. et al. Rho GTPase regulation of α-synuclein and VMAT2: implications for pathogenesis of Parkinson’s disease. *Mol. Cell. Neurosci.* **48**, 29–37 (2011). 10.1016/j.mcn.2011.06.002 PMC3163163 21699982

[18] Hussain, N. K. et al. Endocytic protein intersectin-l regulates actin assembly via Cdc42 and N-WASP. *Nat. Cell Biol.* **3**, 927–932 (2001). 10.1038/ncb1001-927 11584276

[19] Zhang, Y., James, M., Middleton, F. A. & Davis, R. L. Transcriptional analysis of multiple brain regions in Parkinson’s disease supports the involvement of specific protein processing, energy metabolism, and signaling pathways, and suggests novel disease mechanisms. *Am. J. Med. Genet. Part B Neuropsychiatr. Genet.* **137B**, 5–16 (2005). 10.1002/ajmg.b.30195 15965975

[20] Ying, L. et al. Regulation of Cdc42 signaling by the dopamine D2 receptor in a mouse model of Parkinson’s disease. *Aging Cell* **21**, e13588 (2022). 10.1111/acel.13588 PMC9124300 35415964

[21] Schnack, C., Danzer, K. M., Hengerer, B. & Gillardon, F. Protein array analysis of oligomerization-induced changes in alpha-synuclein protein–protein interactions points to an interference with Cdc42 effector proteins. *Neuroscience* **154**, 1450–1457 (2008). 10.1016/j.neuroscience.2008.02.049 18541383

[22] Luo, L. RHO GTPASES in neuronal morphogenesis. *Nat. Rev. Neurosci.* **1**, 173–180 (2000). 10.1038/35044547 11257905

[23] Bycroft, C. et al. The UK Biobank resource with deep phenotyping and genomic data. *Nature* **562**, 203–209 (2018). 10.1038/s41586-018-0579-z PMC6786975 30305743

[24] Gudbjartsson, D. F. et al. Large-scale whole-genome sequencing of the Icelandic population. *Nat. Genet.* **47**, 435–444 (2015). 10.1038/ng.3247 25807286

[25] Jónsson, H. et al. Data descriptor: whole genome characterization of sequence diversity of 15,220 Icelanders. *Sci. Data* **4**, 1–9 (2017). 10.1038/sdata.2017.115 PMC5607473 28933420

[26] Eggertsson, H. P. et al. Graphtyper enables population-scale genotyping using pangenome graphs. *Nat. Genet.* **49**, 1654–1660 (2017). 10.1038/ng.3964 28945251

[27] Eggertsson, H. P. et al. GraphTyper2 enables population-scale genotyping of structural variation using pangenome graphs. *Nat. Commun.* **10**, 5402 (2019). 10.1038/s41467-019-13341-9 PMC6881350 31776332

[28] Kong, A. et al. Detection of sharing by descent, long-range phasing and haplotype imputation. *Nat. Genet.* **40**, 1068–1075 (2008). 10.1038/ng.216 PMC4540081 19165921

[29] Gudbjartsson, D. F. et al. Sequence variants from whole genome sequencing a large group of Icelanders. *Sci. Data* **2**, 1–11 (2015). 10.1038/sdata.2015.11 PMC4413226 25977816

[30] Halldorsson, B. V. et al. The sequences of 150,119 genomes in the UK Biobank. *Nature* **607**, 732–740 (2022). 10.1038/s41586-022-04965-x PMC9329122 35859178

[31] McLaren, W. et al. The Ensembl variant effect predictor. *Genome Biol.* **17**, 1–14 (2016). 10.1186/s13059-016-0974-4 PMC4893825 27268795

[32] Bulik-Sullivan, B. et al. LD score regression distinguishes confounding from polygenicity in genome-wide association studies. *Nat. Genet.* **47**, 291–295 (2015). 10.1038/ng.3211 PMC4495769 25642630

[33] Mantel, N. & Haenszel, W. Statistical aspects of the analysis of data from retrospective studies of disease. *J. Natl Cancer Inst.* **22**, 719–748 (1959). 13655060

[34] Iwaki, H. et al. Accelerating medicines partnership: Parkinson’s disease. genetic resource. *Mov. Disord.* **36**, 1795 (2021). 10.1002/mds.28549 PMC8453903 33960523

