# Supplementary material for: Loss-of-function variants in ITSN1 confer high risk of Parkinson’s disease
Source: NPJ Parkinsons Dis. 2024 Aug 15;10:140. doi: 10.1038/s41531-024-00752-9 (PMC11327306; doi:10.1038/s41531-024-00752-9)

Supplementary Information

Loss-of-function variants in *ITSN1* confer high risk of Parkinson‘s disease

Astros Th. Skuladottir, Vinicius Tragante, Gardar Sveinbjornsson, Hannes Helgason, Arni Sturluson, Anna Bjornsdottir, Palmi Jonsson, Vala Palmadottir, Olafur A. Sveinsson, Brynjar O. Jensson, Sigurjon A. Gudjonsson, Erna V. Ivarsdottir, Rosa S. Gisladottir, Arni F. Gunnarsson, G. Bragi Walters, Gudrun A. Jonsdottir, Thorgeir E. Thorgeirsson, Gyda Bjornsdottir, Hilma Holm, Daniel F. Gudbjartsson, Patrick Sulem, Hreinn Stefansson, and Kari Stefansson

Supplementary Figure 1. Manhattan and QQ plot

Plots showing the association results from the gene-based LOF burden test for PD; a) Manhattan plot where the -$\log_{10}P$-values (y-axis) are plotted for each gene against their chromosomal position (x-axis) and b) QQ plot where the observed the -$\log_{10}P$-values (y-axis) are plotted for each gene against the theoretical distribution (red line, x-axis). The blue line represents the significance threshold (4.2×10^{-6}).

a

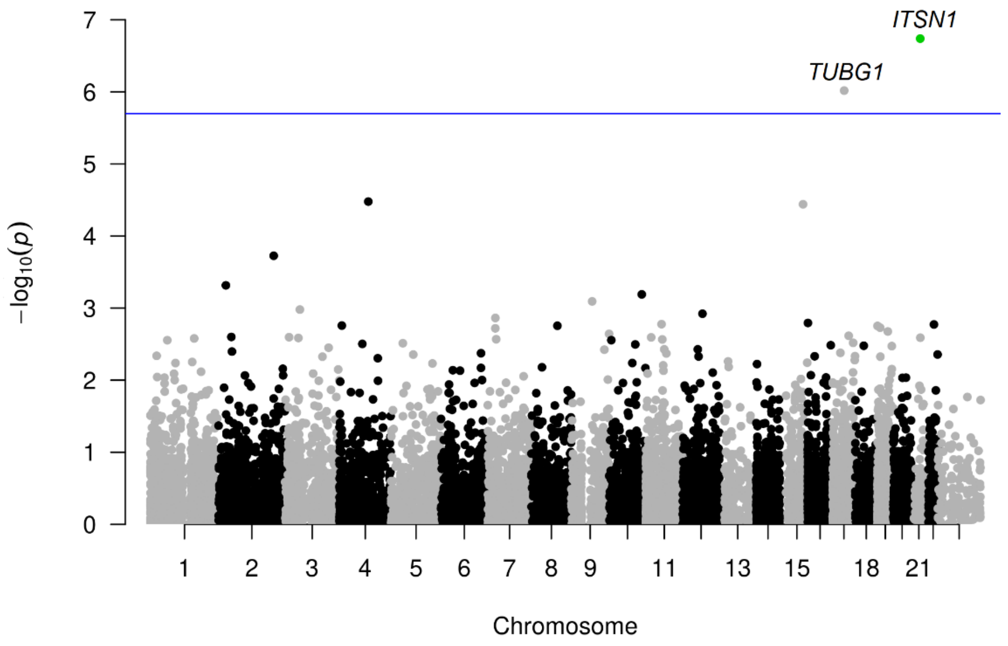

b

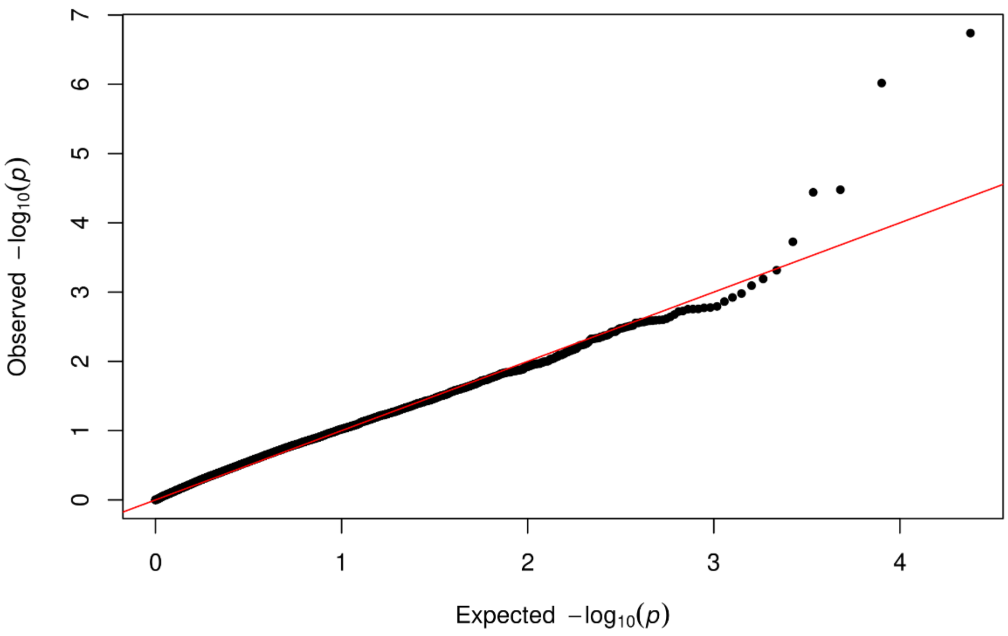

Supplement: Supplementary file 1 — Supplementary Figure [file 41531_2024_752_MOESM1_ESM.pdf]
